# Atrial fibrillation: effects beyond the atrium?

**DOI:** 10.1093/cvr/cvv001

**Published:** 2015-01-13

**Authors:** Rohan S. Wijesurendra, Barbara Casadei

**Affiliations:** Division of Cardiovascular Medicine, BHF Centre of Research Excellence, University of Oxford, John Radcliffe Hospital, Level 6 West Wing, Oxford OX3 9DU, UK

**Keywords:** Atrial fibrillation, Ventricular function, Ventricular structure

## Abstract

Atrial fibrillation (AF) is the most common sustained clinical arrhythmia and is associated with significant morbidity, mostly secondary to heart failure and stroke, and an estimated two-fold increase in premature death. Efforts to increase our understanding of AF and its complications have focused on unravelling the mechanisms of electrical and structural remodelling of the atrial myocardium. Yet, it is increasingly recognized that AF is more than an atrial disease, being associated with systemic inflammation, endothelial dysfunction, and adverse effects on the structure and function of the left ventricular myocardium that may be prognostically important. Here, we review the molecular and *in vivo* evidence that underpins current knowledge regarding the effects of human or experimental AF on the ventricular myocardium. Potential mechanisms are explored including diffuse ventricular fibrosis, focal myocardial scarring, and impaired myocardial perfusion and perfusion reserve. The complex relationship between AF, systemic inflammation, as well as endothelial/microvascular dysfunction and the effects of AF on ventricular calcium handling and oxidative stress are also addressed. Finally, consideration is given to the clinical implications of these observations and concepts, with particular reference to rate vs. rhythm control.

## 1. Introduction

Atrial fibrillation (AF) is the most common sustained clinical arrhythmia and its incidence is strongly associated with increasing age^1^ and comorbidity,^2^ although up to a third of cases occur in the absence of identifiable underlying cardiovascular disease and have been termed ‘lone’ AF.^2^ Regardless of aetiology, AF is associated with significant morbidity and an estimated two-fold increase in premature mortality.^3^

The discovery that AF triggers are located in the pulmonary veins in the majority of cases led to the development of pulmonary vein isolation via catheter ablation,^4^ now a widely used and effective clinical treatment for paroxysmal AF.^5^ Persistent AF presents a greater challenge to successful catheter ablation, due to more advanced electrical and structural atrial remodelling. Efforts to increase our pathophysiological understanding of AF have focused on unravelling the mechanisms responsible for these progressive atrial abnormalities.^6^ However, it is now increasingly recognized that AF can also adversely affect *ventricular* function.^7^ While some patients develop overt left ventricular (LV) dysfunction and heart failure, others present with subclinical alterations in ventricular structure and function, which may only be revealed by detailed investigation.^8,9^

Ventricular remodelling may be contributed to by the adverse haemodynamic effect of loss of coordinated atrial contraction and/or tachycardia-mediated cardiomyopathy from a persistently elevated ventricular rate. The possibility of additional mechanisms is suggested by the observation that some patients with AF and a controlled ventricular rate develop abnormalities in ventricular function, which are reversible following restoration of sinus rhythm (SR).^10,11^ One such potential mechanism is impaired myocardial perfusion and perfusion reserve due to microvascular coronary dysfunction in the presence of AF.^12^ This could underlie the clinical observation of ischaemic chest pain, ST depression, and troponin release in patients with AF and no obstructive epicardial coronary artery disease.^13,14^

In addition to the LV abnormalities described, AF is also associated with systemic inflammation and impaired endothelial function.^15–17^ A deeper understanding of the relationships between inflammation, endothelial function, and ventricular function in AF and the reversibility or otherwise of these alterations following restoration of SR may lead to more rational therapeutic strategies and ultimately to improved outcomes for patients.

**Figure 1:**
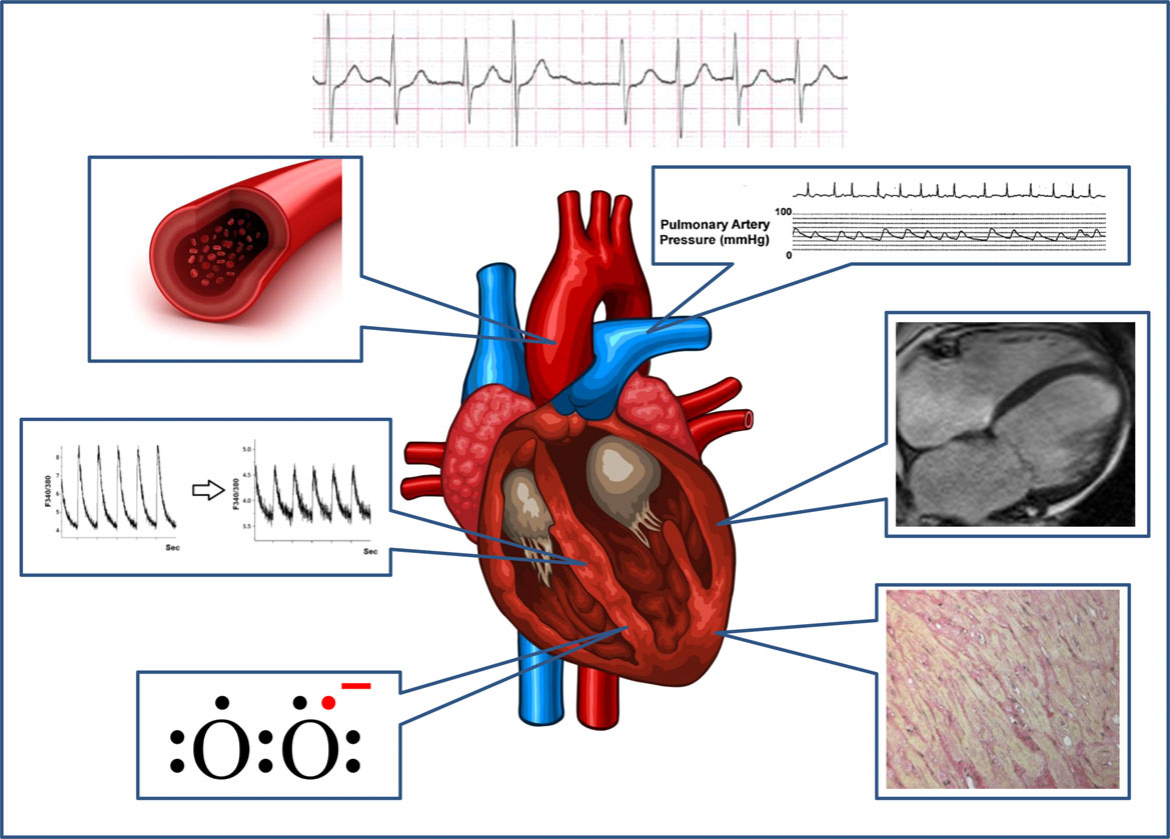
Systemic effects of AF. Clockwise from top right: adverse haemodynamic effects; ventricular remodelling and dysfunction; diffuse and focal myocardial fibrosis; ventricular oxidative stress; impaired ventricular calcium handling; inflammation and impaired endothelial function, myocardial perfusion, and perfusion reserve. Top right image reproduced with permission from ref.^18^ Bottom right image reproduced with permission from ref.^19^ Middle left image reproduced with permission from ref.^20.^

Here, we review the systemic correlates of AF (*Figure 1*) with a focus on AF-associated changes in ventricular structure, perfusion, and overall function. The relationship between AF and systemic inflammation and endothelial dysfunction is also briefly addressed. Finally, we discuss the implications of these findings for preclinical and basic research and provide a current clinical perspective.

## 2. AF and impaired LV function: the chicken or the egg?

AF is associated with impaired ventricular function, with a spectrum of severity ranging from congestive heart failure to subtle subclinical alterations in LV performance. Causation is much more challenging to ascertain, particularly when the index presentation is with both AF and LV dysfunction. The possibilities are that AF may promote ventricular impairment by raising the ventricular rate (the so-called tachycardia-induced cardiomyopathy), or by other mechanisms such as endothelial dysfunction and impaired myocardial perfusion. Alternatively, a pre-existing ventricular cardiomyopathy may lead to AF, via mechanical stretch and structural remodelling of the atria and altered myocardial intracellular calcium handling.^21^ Finally, AF and LV dysfunction may be the culmination of common cardiovascular conditions such as hypertension or coronary artery disease, and could be considered ‘chamber-specific expressions’ of global myocardial damage.^21^ Irrespective of causality, AF is common in heart failure, with a reported prevalence ranging from 13 to 27%.^22^ It is also associated with heart failure severity—up to 50% of patients with severe heart failure are affected by AF.^23^ Furthermore, AF is clearly associated with adverse prognosis (including premature mortality) in heart failure trials,^24–26^ although it remains controversial whether this effect is truly independent.^22^

Studies in a canine heart failure model have demonstrated that AF induced by a primary impairment in LV function is not associated with characteristic atrial electrical abnormalities such as the reduced atrial refractory period seen in AF induced by rapid atrial pacing with a controlled ventricular response.^27^ Instead, a substantial increase in the heterogeneity of conduction during atrial pacing is noted due to extensive atrial interstitial fibrosis, a finding not seen following short-term rapid atrial pacing.^27^ In clinical practice, a significant improvement in ventricular function following reduction in AF burden by cardioversion or ablation is perhaps the best identifier of a causal role of AF in the associated LV dysfunction, demonstrating that ventricular impairment is a reversible secondary effect. This notwithstanding, there are often multiple interacting causative factors in human AF and current classification schemes are unable to capture the inherent complexity and heterogeneity. This complicates the study of human AF, and underlines the importance of translating findings from animal models, each of which partially reproduces the pathophysiological spectrum of clinical AF.^28^

## 3. Challenges to the study of ventricular abnormalities in human AF

The systematic evaluation of ventricular molecular and cellular abnormalities in AF is limited by poor access to human tissue samples. Atrial cardiomyocytes or trabeculae can be isolated from samples of the right atrial appendage excised at the time of cardiac surgery and cardiopulmonary bypass. Although the results of *in vitro* experiments on such preparations may not be fully reflective of *in vivo* conditions, these investigations have provided relevant insights into the changes in ion channel expression and function that accompany human AF.^29^

The situation is even more challenging for investigations of the ventricular myocardium, as ventricular biopsy is rarely clinically indicated and is difficult to justify purely for research purposes due to the risk of complications.^30^ Even when undertaken, biopsy carries the risk of sampling error, and patchy abnormalities may remain undetected.^31^ The limited human data in the literature largely represent small case series of ventricular biopsy^32^ or studies of tissue from explanted hearts following cardiac transplant.^20^ Interpretation of these data is made more difficult by the lack of matched tissue from healthy control subjects.

## 4. LV myocardial changes associated with AF

### 4.1 Fibrosis

Although human data reporting ventricular changes in the presence of AF are sparse, one series that investigated 14 young patients with apparent ‘lone’ AF refractory to initial medical therapy demonstrated abnormal histology in all cases.^32^ Importantly, all patients had structurally normal hearts without atrial or ventricular dilatation and a normal LV ejection fraction. Three of 14 had evidence of focal myocarditis (eosinophilic or lymphocytic) and initiation of corticosteroid therapy in addition to antiarrhythmic medications induced recovery of SR in all of this group. A further three patients had evidence of a diffuse cardiomyopathic process, whereas the remaining eight patients had a non-specific diffuse fibrotic change with or without evidence of cardiomyocyte necrosis. However, such changes may not be specific to AF as a separate study documented similar histological findings in the majority of patients with other forms of supraventricular tachycardia.^33^

More data are available from non-invasive studies using MRI techniques, which have aimed to identify ventricular fibrosis in AF. Diffuse fibrosis cannot be visualized by the widely used method of late gadolinium enhancement (LGE) cardiac MRI, which relies on the presence of areas of normal myocardium to identify discrete areas of focal fibrosis, scarring, or infarction. However, diffuse fibrosis can be detected by the recently developed methods of *T*_1_ mapping,^34^ usually requiring administration of a gadolinium-based contrast agent. Several methods have been described: some rely on *T*_1_ measurement following contrast administration alone, while others combine these data with native (i.e. pre-contrast) *T*_1_ measurement and haematocrit to define myocardial volume of distribution or myocardial extracellular volume fraction (ECV).^35,36^ Contrast diffuses freely in the extracellular space and a low post-contrast *T*_1_ value reflects an elevated ECV, which is usually indicative of diffuse fibrosis. This method has been validated against histological assessment of fibrosis in right ventricular endomyocardial biopsy specimens taken from subjects who had undergone orthotopic heart transplantation.^19^ A study on post-contrast *T*_1_ mapping in subjects with paroxysmal (*n* = 40) and persistent AF (*n* = 27) as well as healthy controls (*n* = 23) demonstrated significantly lower post-contrast *T*_1_ values in both sets of AF patients compared with age- and sex-matched controls.^37^ Additionally, patients with persistent AF had lower post-contrast *T*_1_ values than the paroxysmal AF group. In keeping with established data showing that diffuse myocardial fibrosis increases with age, there was a negative correlation between increasing age and post-contrast *T*_1_ value in the healthy controls. In a multivariate model, only age, AF class, and LV ejection fraction were independent correlates of post-contrast ventricular *T*_1_ values. These data suggest that increasing AF burden is associated with progressive diffuse LV fibrosis, and may play a role in adverse cardiac remodelling.

**Figure 2:**
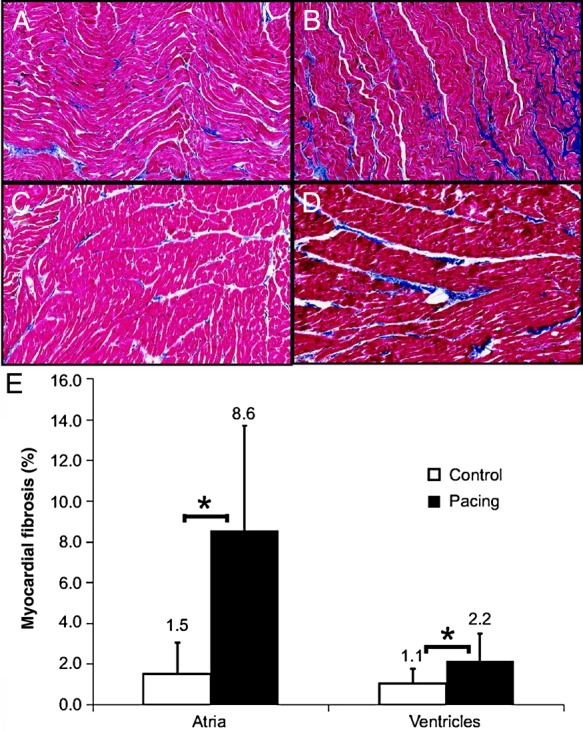
Fibrosis in the dog at baseline and at 6 months of AF. (*A*) Left atrial (LA) sample from a control dog (2.0% fibrosis). (*B*) LA sample from a 6-month AF dog (10.1% fibrosis). (*C*) LV sample from a control dog (1.0% fibrosis). (*D*) LV sample from a 6-month AF dog (2.4% fibrosis). (*E*) Fibrosis levels increased in both the atria and ventricles during chronic AF. Values above bars indicate SDs. *P < 0.01. Reproduced with permission from ref.^39^

Data from animal studies suggest that the development of LV fibrosis is related to an elevated ventricular rate in the presence of AF. Induction of AF by rapid atrial pacing in dogs in the presence of atrioventricular (AV) node ablation and a paced ventricular rate of 80 bpm was sufficient to induce atrial but not ventricular fibrosis, whereas dogs without AV node ablation and a more rapid ventricular rate did develop significant ventricular fibrosis.^38^ Heart failure and ventricular fibrosis have been reported in a similar dog model, despite the use of oral digoxin and metoprolol with the aim of achieving ventricular rate control.^39^ However, the degree of ventricular fibrosis was less marked than the observed atrial fibrosis (*Figure 2*), and the ventricular rates of up to 180 bpm reported in this study may have contributed to a tachycardia-mediated cardiomyopathy; no corresponding increase in ventricular fibrosis was detected by the same investigators in a pig model.^39^

Findings consistent with the paradigm of tachycardia-induced ventricular fibrosis have also been described in patients who had undergone previous ablation for incessant focal atrial tachycardia.^40^ Focal atrial tachycardia provides a good model for the study of human atrial arrhythmia as long-term ‘cure’, and maintenance of SR is feasible following ablation. The subgroup with LV cardiomyopathy at baseline had lower post-contrast *T*_1_ values on CMR imaging at a mean of 5 years following ablation, indicating a greater burden of diffuse ventricular fibrosis that was irreversible despite restoration of normal SR and improvement in LV ejection fraction. Interestingly, there was no significant difference in mean heart rate on 24-h ECG monitoring prior to ablation between the patients with and without cardiomyopathy. While this may reflect the lack of statistical power to detect a true difference, it is also possible that the maximum ventricular rate tolerated before the development of ventricular fibrosis and impaired LV function varies between individuals.

Atrial and ventricular fibrosis in response to AF may be regulated by different mechanisms. The study of cultured rat atrial and ventricular fibroblasts shows that NADPH oxidase 4 (NOX-4) is more highly expressed in the atria than the ventricles, and this mediates the enhanced collagen and fibronectin production of atrial fibroblasts compared with ventricular fibroblasts in response to TGF-β.^41^ Thus, ventricular tissue appears inherently less susceptible to the development of fibrosis than atrial tissue, though prolonged exposure to an elevated ventricular rate can nevertheless still result in ventricular fibrosis. In summary, although it is currently not possible to definitively determine causality, there is strong circumstantial evidence that AF can induce diffuse ventricular fibrosis.^42^

Furthermore, a recent study emphasized the prevalence and prognostic significance of focal myocardial scar, as detected by LGE. LV LGE was present in 13% of patients with AF and without known prior myocardial infarction undergoing a routine cardiac MRI study prior to pulmonary vein isolation.^43^ Intriguingly, the presence of LGE was a powerful predictor of mortality with each 1% increase in LGE associated with a 15% increased risk of death. The patterns of LGE seen in this study included an ischaemic pattern, which probably reflects silent myocardial infarction, and a non-ischaemic pattern, which may relate to inclusion of patients with heart failure and/or reduced ejection fraction or may reflect the presence of an AF-related ventricular cardiomyopathy in some of the studied cohort. The presence of focal ventricular fibrosis in patients with AF is not well described, and further studies are needed to identify whether these findings are more prevalent in this population than in a matched cohort of patients in SR.

There are also data defining the prognostic value of diffuse LV fibrosis in AF. ECV expansion (mainly due to diffuse fibrosis) is associated with increased mortality and heart failure in broadly unselected patients undergoing clinical cardiac MRI independently of age, ejection fraction, and previous infarct size,^44^ and also strongly predicts recurrence of AF following catheter ablation in hypertensive patients.^45^ If AF caused diffuse LV fibrosis and adverse remodelling, targeting an aggressive rhythm control strategy to the subgroup of patients in whom AF is the primary pathology (rather than a consequence of another disease state) could clearly be important. The hypothesis that this strategy would improve clinical outcomes would need to be examined in an appropriately powered clinical trial.

### 4.2 Perfusion and perfusion reserve

Patients with AF and without obstructive epicardial coronary artery disease may still demonstrate clinical evidence of ischaemia, such as chest pain, ST depression, or troponin release, often at the onset of arrhythmia,^13,14^ implying the presence of either microvascular coronary dysfunction or arrhythmia-induced coronary artery spasm. In a pig pacing model of AF, 6 h of rapid atrial pacing (600 bpm) are sufficient to elicit a substantial reduction in coronary flow reserve (CFR) in the absence of any significant change in fractional flow reserve (FFR).^46^ CFR is influenced by abnormal flow in the epicardial coronary arteries as well as the microcirculation, whereas FFR is specific to epicardial disease; hence, these data support the hypothesis that AF can rapidly adversely affect microvascular coronary function. The investigators report that this reduction in CFR was abolished by pretreatment with dronedarone (but not amiodarone), and suggest that this may reflect an attenuating effect of dronedarone on oxidative stress and consequent microvascular flow abnormalities.^46^ However, as ventricular rates were not reported, it is not possible to identify the extent to which tachycardia (and reduced diastole duration) may have contributed to the reduction in CFR.

**Figure 3:**
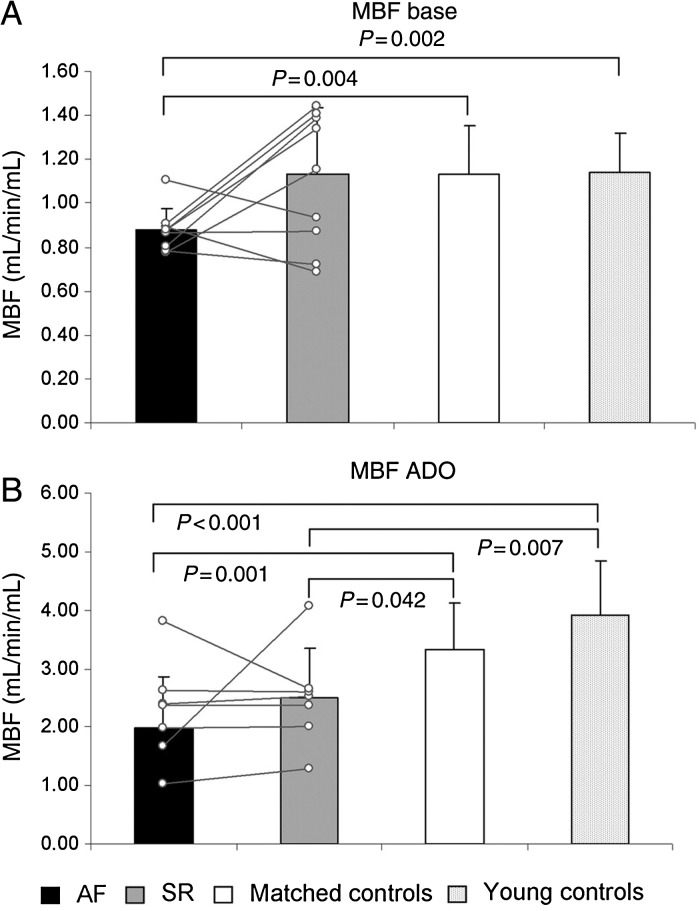
Myocardial perfusion and perfusion reserve. Myocardial blood flow in 10 patients (mean and individual trends) by PET during AF and during SR 4.1 ± 2.3 months after cardioversion (SR) in comparison with age-/risk-matched and young controls as measured at baseline (*A*) and under adenosine (*B*) for assessment of perfusion reserve. Reproduced with permission from ref.^12^

Myocardial perfusion and perfusion reserve have been evaluated using PET and radioactively labelled water in patients with persistent AF after exclusion of epicardial coronary artery disease.^12^ Myocardial blood flow at rest was markedly reduced in the AF group compared with age- and risk-matched controls as was adenosine-induced hyperaemic flow (*Figure 3*). Minimal coronary vascular resistance at rest and under hyperaemia was also significantly increased in AF patients compared with controls. Finally, in the subgroup of patients that maintained SR following cardioversion, myocardial blood flow at rest normalized and hyperaemic flow and coronary vascular resistance were significantly improved from baseline. These human findings are in accordance with the animal data, and suggest that the presence of AF induces abnormalities in microvascular coronary function that are at least partially reversible with restoration of SR. However, the subgroup that maintained SR following cardioversion was small (10 patients), and confirmation of these findings in a larger cohort is awaited.

In a separate study, the effect of AF on coronary flow, flow reserve, and vascular resistance was assessed invasively in patients in SR with no ventricular or valvular dysfunction, and no significant epicardial coronary artery disease.^47^ A coronary Doppler catheter was placed and measurements taken in SR, during AF induced by bursts of atrial pacing, and during regular atrial tachycardia with a rate of pacing equal to the overall ventricular rate observed during AF. The relative contribution of the irregularity of ventricular contraction in AF was also assessed by the inclusion of a second group of patients with complete AV block and dual-chamber pacemakers *in situ*; in this group, the ventricle was paced regularly at 115 bpm before and after induction of AF. Acute induction of AF led to an increase in coronary flow, which was lower than that seen in the presence of rapid atrial pacing and was associated with a reduction in CFR and vascular resistance. In contrast, only a small effect on coronary flow and an increase in CFR were seen in the group with pacemakers *in situ*. The increase in coronary flow following induction of AF is likely to be explained by the elevated ventricular rate following acute induction of AF, as patients in this study were not receiving rate control medications. However, this absolute rise in coronary flow appears insufficient relative to the increase in ventricular rate and a consequent increase in myocardial metabolic demand. A study in pigs demonstrated a similar finding of ‘supply-demand ischaemia’ in the atria following acute induction of AF,^48^ evidenced by an increase in atrial lactate production despite compensatory rises in both atrial coronary conductance and oxygen extraction.

Atrial supply-demand ischaemia has also been proposed as a potential trigger for atrial structural remodelling and has a molecular phenotype that resembles changes seen in hibernating ventricular myocardium.^48^ Indeed, chronic ventricular ischaemia could underlie the impaired ventricular function commonly seen in AF.

### 4.3 Microvascular/endothelial dysfunction and inflammation

**Figure 4:**
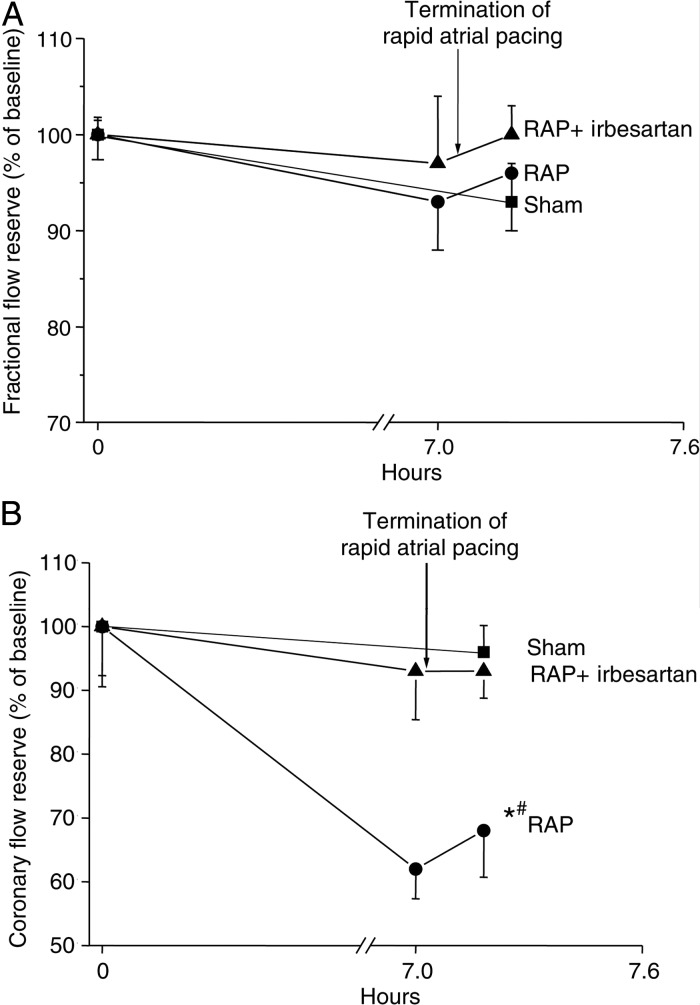
Flow reserve measurements during 7 h of rapid atrial pacing (RAP) with and without administration of irbesartan. (*A*) RAP has no effect on FFR compared with sham animals, suggesting that RAP does not affect blood flow in the epicardial coronary arteries (mean ± SEM). (*B*) RAP leads to a reduction in CFR compared with sham (*P = 0.009, Tukey test) and RAP with irbesartan (^#^*P* = 0.022, Tukey test). Reproduced with permission from ref.^49^

The irregular pulse interval that is typical of AF seems to play a role in the impaired coronary flow observed in these patients, even when the overall ventricular rate is well controlled. Irregular pulse intervals can adversely affect blood pressure, but there was no correlation between blood pressure and coronary flow in the invasive study discussed,^47^ leaving the possibility that irregular ventricular contraction might cause coronary vasoconstriction, possibly by increasing the release of vasoactive peptides such as angiotensin II or endothelin. Studies in a pig model of rapid atrial pacing have suggested that the angiotensin receptor blocker, irbesartan, abolishes the impairment of microvascular flow otherwise seen with rapid atrial pacing (*Figure 4*), as well as preventing ventricular oxidative stress.^49^ The link between AF and impaired vascular function is not limited to the coronary circulation, and there are numerous reports confirming an association between AF and systemic endothelial dysfunction. Non-invasive assessment of endothelial function is most readily undertaken using flow-mediated dilatation (FMD), which assesses nitric oxide production in response to shear stress and by measurement of plasma levels of markers of endothelial damage or dysfunction, such as the von Willebrand factor and circulating endothelial cells. Endothelium-dependent FMD is abnormal in patients with AF,^15^ and a causative role for AF in endothelial dysfunction is suggested by the finding that cardioversion^50–52^ and successful catheter ablation of AF^16^ are associated with significant improvements in endothelial function.

Inflammation represents a potential link between AF, impaired endothelial function, and a reduction in myocardial perfusion and perfusion reserve. A recent study investigated the acute effects of AF induced by burst atrial pacing immediately prior to catheter ablation in patients with a history of paroxysmal AF but in SR at the time of the procedure.^53^ This group was compared with patients who underwent regular atrial pacing at 150 bpm, a control group of patients with paroxysmal AF who were not paced and remained in SR, and a further control group of patients with no history of AF undergoing ablation of left-sided accessory pathways who were also not paced and in SR. The study showed that even at the very short time point of 15 min, rapid atrial rates (either through AF or rapid atrial pacing) were associated with increased local platelet activation and thrombin generation. Furthermore, AF (but not rapid atrial pacing) additionally led to local and systemic endothelial dysfunction (as assessed by levels of asymmetric dimethylarginine) and inflammation (as assessed by levels of soluble CD40L). These data suggest that even a short duration of AF may cause inflammation and endothelial dysfunction. Further studies are needed to investigate whether this may be an important mechanism underlying impaired myocardial perfusion and perfusion reserve and the thromboembolic risk of asymptomatic short-lasting episodes of AF.^54^

### 4.4 Ventricular remodelling and function

It is well recognized that AF and other supraventricular tachycardias with an uncontrolled ventricular rate can cause a transient and reversible ventricular dysfunction that is termed ‘tachycardia-induced cardiomyopathy’.^55,56^ More recently, data from animal and human studies have suggested that, even in the absence of persistently elevated ventricular rates, AF can be associated with adverse atrial and ventricular remodelling and impairment of LV systolic and diastolic function.^10,11,39^ The relationship between AF and LV dysfunction is complex and potentially bi-directional, leading to a vicious electromechanical cycle.^7^ Causality in individual cases cannot be determined at a single time point, but a number of studies have undertaken serial evaluation around an intervention aimed at maintaining SR, and these have provided an important insight into causal relationships in the studied cohorts.

A recent randomized study of patients with persistent AF, symptomatic heart failure, and LV ejection fraction <50% demonstrated a significant improvement in LV ejection fraction by 1 month (and persisting at 6 months) in those randomized to catheter ablation (*n* = 26) compared with those randomized to medical rate control (*n* = 24).^10^ A similar improvement was seen in peak oxygen consumption and was mirrored by reductions in plasma B-type natriuretic peptide concentrations and NYHA class. Importantly, all patients were assessed for adequate rate control at baseline by ambulatory monitoring and exercise testing, thereby demonstrating that even rate-controlled AF has deleterious effects on LV function. The improvement in LV ejection fraction shown in this study is in keeping with the results of other (mainly observational) studies of patients with AF undergoing catheter ablation (summarized in a recent meta-analysis^57^).

A study involving serial echocardiographic studies prior to and at regular intervals up to 1 year after radiofrequency ablation demonstrated that ventricular function is not entirely normal, even in patients with apparent lone AF.^8^ This carefully selected group demonstrated echocardiographic evidence of subtle LV systolic and diastolic dysfunction, compared with age- and sex-matched controls, although LV ejection fraction remained largely within the normal range. The exclusion of other causes of LV dysfunction strongly suggests that AF was causally associated with these abnormalities. Further evidence for this is provided by the post-ablation follow-up echocardiograms, which demonstrated a progressive improvement in LV performance over time. Finally, there was a dose–response relationship between AF burden and adverse ventricular function with more marked baseline impairment and subsequent improvement noted in the chronic AF group than in the paroxysmal AF group.

**Figure 5:**
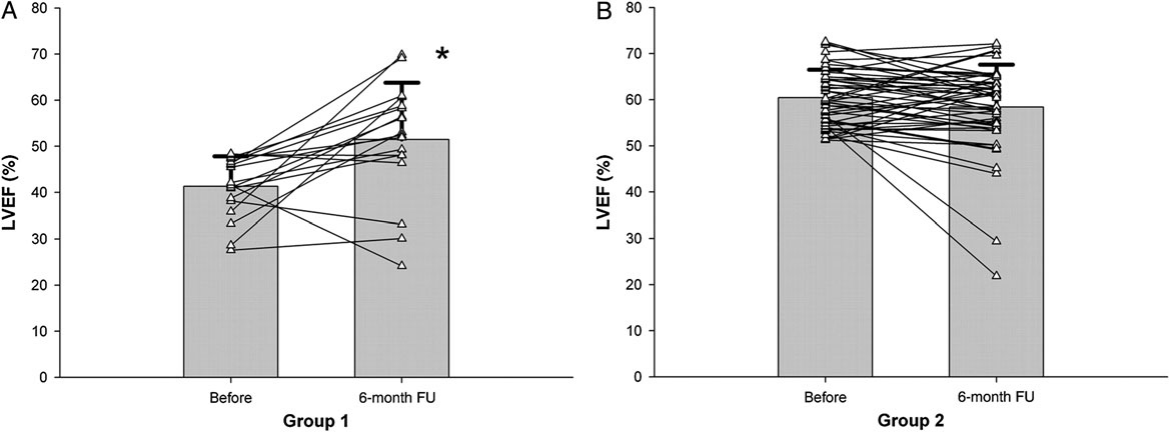
Effect of a pulmonary vein isolation ablation procedure on LV ejection fraction (LVEF). (*A*) Group 1 (LVEF <50% at baseline) demonstrates a significant increase in LVEF at 6 months post-procedure (*P = 0.004). (*B*) There is no significant difference in LVEF between baseline and 6 months in Group 2 (LVEF ≥50% at baseline). Of note, all six Group 2 patients with an LVEF reduction after the ablation and the only patient in Group 1 who experienced a significant decrease in LV function following PVI demonstrated AF recurrences. Reproduced with permission from ref.^58^

A subsequent study which used cardiac MRI to evaluate LV function before and after ablation for short-lasting paroxysmal AF confirmed impaired LV systolic function at baseline and improvement in LV ejection fraction following ablation in all but patients with a normal ejection fraction at baseline (*Figure 5*).^58^ The findings in the latter group may reflect the relatively low AF burden and the poor sensitivity of ejection fraction for detecting subtle changes in LV systolic function. Support for the latter possibility comes from a study by Tops *et al.*^59^ who utilized speckle-tracking to investigate LV strain in selected patients undergoing ablation for AF. Longitudinal and circumferential LV strain and strain rate were impaired at baseline compared with healthy controls. Intriguingly, maintenance of SR at follow-up was associated with improvement in these parameters, whereas recurrence of AF was associated with further deterioration. These data support the presence of subtle LV dysfunction in patients with AF and maintained LV ejection fraction; its reversibility following AF ablation strongly implies that AF is playing a causal role.

### 4.5 Haemodynamics

AF is associated with impaired haemodynamic parameters such as reduced cardiac output and elevated pulmonary artery pressures. Multiple mechanisms may contribute to these findings including (i) loss of atrial systole and AV synchrony, (ii) elevated overall ventricular rate, (iii) irregularity in the pulse interval, (iv) valvular regurgitation, and (v) neurohormonal effects. The role of some of these mechanisms has been directly assessed in human studies. In particular, in an invasive study involving ventricular pacing in patients with AF undergoing AV node ablation, AF and all forms of pacing were associated with reduced cardiac output compared with SR, emphasizing the importance of AV synchrony.^18^ Additionally, the effect of irregularity of the pulse interval was assessed by comparison of irregular versus regular ventricular stimulation. This assessment was undertaken after AV node ablation, with the rate of ventricular stimulation individualized to each patient based on the ventricular rate that had been observed in intrinsically conducted AF immediately prior to AV node ablation. Importantly, cardiac output was significantly reduced, and pulmonary capillary wedge and right atrial pressures were significantly increased in the presence of an irregular pulse interval compared with regular pacing at the same rate. These results are in agreement with those of a prior similar study,^60^ and represent direct *in vivo* evidence of the adverse haemodynamic consequences of an irregular pulse interval *per se*.

A separate study showed that, in patients with persistent AF and impaired LV function, improvement of atrial and ventricular function after successful cardioversion each follows a different time course.^61^ While heart rate is slowed immediately and atrial contractility recovers by 1 week and remains unchanged thereafter, recovery of LV systolic function takes 1 month. Improvements in exercise tolerance and peak oxygen consumption mirrored the time course of recovery in LV function. These data suggest that the impairment in LV function seen in persistent AF may be due to a transient ventricular cardiomyopathy or stunning, due in part to an elevated ventricular rate but perhaps also to the irregular pulse interval.

## 5. Molecular and cellular changes in the LV myocardium associated with AF

The molecular and cellular effects of AF on the ventricular myocardium are challenging to the study in humans due to limited clinical indications for ventricular biopsy and few other means of accessing ventricular tissue. In comparison, it is relatively straightforward to assess atrial tissue that is frequently excised in common cardiothoracic operations such as on-pump coronary artery bypass grafting. As a result, there are few studies which have explored this area in humans, although several findings have been obtained in animal models of AF.

### 5.1 Oxidative stress

Oxidative stress refers to the chronic disturbance of redox circuits and redox-responsive signal transduction pathways, and atrial oxidative stress is known to play an important pathophysiological role in the induction and maintenance of AF.^62–65^ Less is known regarding the potential importance of ventricular oxidative stress in AF. Limited data are available from a study by Cai *et al.*,^66^ conducted in a pig model of rapid atrial pacing. The protein level of endothelial nitric oxide synthase was significantly decreased in LV tissue from animals with pacing-induced AF compared with controls, although this difference was less pronounced than in the left atrium. Importantly, this finding could not be attributed to an elevated ventricular rate as both groups of animals underwent AV node ablation and the ventricles were paced at 100 bpm in both the AF and control groups. A separate study, also conducted in a pig model of rapid atrial pacing, further demonstrated a significant increase in ventricular NADPH oxidase 2 (NOX2) expression, a finding that was prevented by treatment with irbesartan.^49^ Ventricular concentrations of F_2_-isoprostanes and LOX-1 protein, both markers of oxidative stress, were also elevated by rapid atrial pacing. These observations suggest that rapid atrial pacing induces oxidative stress in LV tissue by an angiotensin-mediated mechanism. Previous work demonstrated that plasma levels of angiotensin II are elevated within 6 h of rapid atrial pacing. Angiotensin II appears to be an important mediator of oxidative stress in AF^67^ by acting via the AT1 receptor to activate mitogen-activated protein kinases and stimulate proliferation of fibroblasts, cellular hypertrophy, and apoptosis as well as inducing calcium release and PKC activation, which in turn promotes activation of NOX2 and NOX1 NADPH-oxidases.^68^ A further study in the same model also reported a reduction in LV oxidative stress in the presence of dronedarone.^46^ In contrast to Cai *et al.*, these studies cannot discriminate between the effect of AF and that of rapid ventricular rates induced by rapid atrial pacing with a competent AV node.

### 5.2 Abnormal calcium handling

Calcium handling abnormalities in the atrial myocardium have been implicated in the initiation and maintenance of AF, and have been recently reviewed.^69^ Briefly, calcium handling abnormalities may be induced by oxidative stress^70^ and can promote AF via (i) induction of delayed afterdepolarizations and triggered events, (ii) enhanced Ca^2+^/calmodulin kinase type 2 activity and associated hyperphosphorylation and increased open probability of the type 2 ryanodine receptor, and (iii) up-regulation of the Na^+^/Ca^2+^ exchanger, increasing the size of the inward current for a given amount of aberrant Ca^2+^ release.^71^

**Figure 6:**
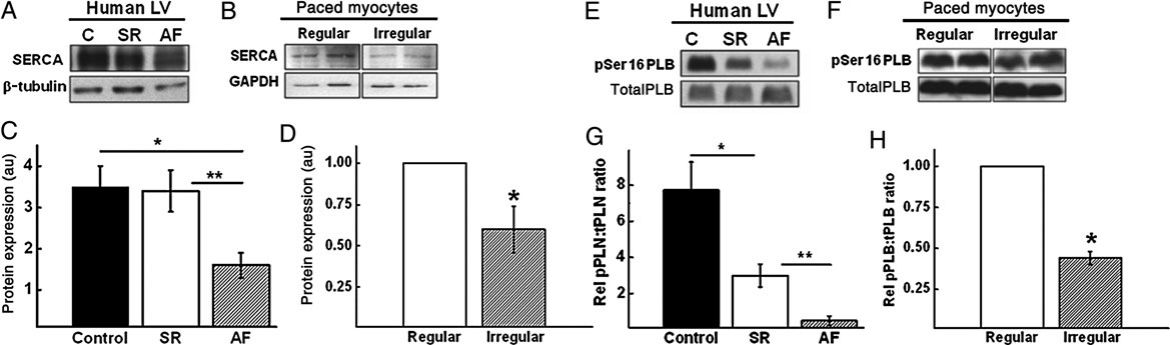
The effect of AF on SERCA and PLB. Representative immunoblots showing SERCA expression in (*A*) human LV myocardium from unused donor heart control (C), heart failure-SR, and heart failure-AF patients; (*B*) regularly paced vs. irregularly paced cardiomyocytes. Quantitative analysis of SERCA expression in (*C*) human myocardial samples (*P* < 0.05 control vs. AF; *P* < 0.01 SR vs. AF) and (*D*) isolated paced cardiomyocytes (*P < 0.05 regular vs. irregular). GAPDH indicates glyceraldehyde-3-phosphate dehydrogenase. Representative immunoblots showing PLB and phosphorylated-PLB expression in (*E*) human LV myocardium from unused donor heart control (C), heart failure-SR, and heart failure-AF patients; (*F*) regularly paced vs. irregularly paced cardiomyocytes. Quantitative analysis of phosphorylated : total PLB expression in (*G*) human myocardial samples (*P < 0.05 control vs. AF; **P < 0.01 SR vs. AF) and (*H*) isolated paced cardiomyocytes (*P < 0.05 regular vs. irregular). pSer, phosphoserine. Reproduced with permission from ref.^20^

In contrast, very sparse data are available on calcium handling in ventricular myocardium in AF. Some insights are provided by a small study which examined human LV myocardial tissue obtained from end-stage heart failure patients with or without AF (*n* = 6 in SR and *n* = 6 in AF) who underwent cardiac transplantation as well as neonatal rat ventricular cardiomyocytes subjected to regular or irregular electrical stimulation *ex vivo*, to simulate SR and AF, respectively (*Figure 6*).^20^ To exclude effects of rate, all cardiomyocytes paced regularly or irregularly were stimulated the same number of times in total over a 24- h period. The mRNA and protein levels of sarcoplasmic reticulum ATPase 2a pump (SERCA), an important determinant of intracellular calcium levels, were significantly lower in the ventricular myocardium of the heart failure patients with AF compared with those in SR. This finding was recapitulated in paced rat ventricular cardiomyocytes, suggesting that irregularity of ventricular contraction influences SERCA expression beyond any effect of heart failure or ventricular rate. Additionally, in both models, AF was associated with a significant decrease in the phosphorylation of phospholamban (PLB), a key regulator of SERCA. Confirmation that these changes affect intracellular calcium handling was obtained from calcium imaging of rat cardiomyocytes, which demonstrated significantly reduced calcium transient amplitude and increased time-to-peak calcium transient amplitude in cardiomyocytes exposed to irregular pacing, compared with unpaced and regularly paced groups. Surprisingly, the rate of decay of the intracellular calcium transient was not significantly different between the groups.

These findings suggest that impaired calcium handling may be partly responsible for the impaired LV contractile function that can be seen in AF, and that this may be influenced by the irregularity of ventricular contraction.

### 5.3 Unanswered questions

Beyond the areas discussed thus far, other molecular and cellular changes have been demonstrated in the atria in AF, but further studies are required to assess if these extend to the ventricle. These include impaired myofibrillar energetics,^72,73^ loss of myofibrils, accumulation of glycogen, changes in mitochondrial shape and size, fragmentation of the sarcoplasmic reticulum, dispersion of nuclear chromatin, and increase in myocyte size.^74^

## 6. Implications for preclinical and basic research

As discussed in the previous sections, experimental evidence suggests that AF has deleterious effects that reach beyond the atrium and affect ventricular structure and function. However, the mechanisms for the observed abnormalities in human and animal ventricular function in AF remain to be conclusively elucidated. Some of the available data appear to implicate abnormalities in ventricular myocytes, including the presence of oxidative stress and impaired calcium handling.

Further studies focussing on ventricular myocytes and ventricular myocardial tissue from animal models of AF are needed to clarify these associations and most importantly to systematically investigate the possibility of a causal relationship between the development of AF and impairment in LV function.

## 7. Clinical perspective

Human studies indicate that control of rhythm with cardioversion or ablation can, at least in selected groups of patients, restore LV function. Of particular importance for the clinician is determining whether this translates into long-term prognostic benefit. Experience over the last decade has not been encouraging: several large randomized clinical trials (including AFFIRM,^75^ RACE,^76^ and AF-CHF^77^) failed to demonstrate any significant improvement in cardiovascular outcomes from a rhythm control strategy over a rate-control strategy. So, is there no rationale for trying to achieve SR in any patient with AF? There are several reasons that this may not be an appropriate conclusion to draw from these data.

First, younger and more symptomatic patients are underrepresented in these trials. This population is more likely to have lone AF and to be at an earlier stage in the natural history of AF, and thus more likely to respond successfully to a rhythm control strategy. It is well established from animal models that the presence of AF induces progressive electrical and structural remodelling that augments AF stability (‘AF-begets-AF’^78^). The corollary of this is that a rhythm control strategy is less likely to successfully maintain SR the longer AF has been established or in disease states where primary atrial structural remodelling is the main substrate for AF (i.e. in the presence of heart failure).

Secondly, the rhythm control strategy employed has relied on antiarrhythmic medications that have limited efficacy and multiple adverse effects. The proportion of patients in the rhythm control arms of these trials who were maintained in SR was relatively modest, ranging from 39^76^ to 63%.^75^ An ‘on-treatment’ analysis of the AFFIRM data suggested that the presence of SR was associated with a lower risk of death.^79^ This suggests that SR either improves survival or is a marker of other factors associated with survival.

It remains to be seen if potentially safer and more effective methods of rhythm control, such as catheter ablation of AF, will improve cardiovascular outcomes compared with rate control or rhythm control with medications alone in selected patient populations. The results of the CABANA trial (ClinicalTrials.gov Identifier NCT00911508), a large randomized trial of 3000 patients that is currently recruiting, will inform whether catheter ablation offers prognostic benefit beyond conventional medical strategy.

## 8. Conclusions

AF is a common disease with significant clinical heterogeneity. Mechanistic and translational studies are providing important insights into its aetiology, and suggest that AF has important adverse effects on LV structure and function, in addition to a complex relationship with systemic inflammation and endothelial function. It is hoped that an improved understanding of the mechanisms underlying these observations will provide a platform for the development of much-needed advances in the therapeutic armamentarium against AF.

**Conflict of interest:** none declared.

## Funding

The authors acknowledge support from the BHF Centre of Research Excellence, Oxford (RE/08/004).
